# Retraction: The Effect of Soluble RAGE on Inhibition of Angiotensin II-Mediated Atherosclerosis in Apolipoprotein E Deficient Mice

**DOI:** 10.1371/journal.pone.0108684

**Published:** 2014-09-18

**Authors:** Dajeong Lee, Kyung Hye Lee, Hyelim Park, Soo Hyuk Kim, Taewon Jin, Soyoung Cho, Ji Hyung Chung, Soyeon Lim, Sungha Park

The authors retract this publication due to concerns about several of the results reported in the article.

After the publication of the article, the authors identified an error in Figure 3A, where the representative oil red stain of the aortic sinus from angiotensin II(-) sRAGE(-) had been duplicated in the angiotensin II(+), sRAGE 2ug image. Upon a review of the data from the article, the authors also identified concerns in relation to possible duplication of data in Figure 5A (RAGE and MCP-1 panels) and Figure 6A (MCP-1 and TNF-α panels). The authors have indicated that the raw data for Figures 3, 5 and 6 are not available to allow for a full evaluation of the concerns noted.

In the light of the concerns about the integrity of the results and the lack of the raw data relevant to the figures affected, the authors and the editors retract this publication.
